# Pediatric autoimmune gastritis: An international, multicentric study

**DOI:** 10.1002/jpn3.70187

**Published:** 2025-08-12

**Authors:** Marco Vincenzo Lenti, Shamim Joudaki, Emanuela Miceli, Edith Lahner, Sara Massironi, Martina Votto, Amelia Licari, Silvia Maria Elena Caimmi, Matteo Bramuzzo, Grazia Di Leo, Marco Crocco, Federica Malerba, Ruggiero Francavilla, Paolo Lionetti, Patrizia Alvisi, Roberto Panceri, Giovanna Zuin, Vincenzo Villanacci, Jacopo Barp, Maurizio Fuoti, Salvatore Oliva, Marina Aloi, Manuela Distante, Amit Assa, Yogev Dotan, Arnaldo Amato, Claudio Romano, Annamaria Staiano, Erasmo Miele, Caterina Strisciuglio, Maria Teresa Fioretti, Fabiana Zingone, Mara Cananzi, Costantino De Giacomo, Giovanni Di Nardo, Andrea Quadrelli, Alessandro Vanoli, Marco Paulli, Bruno Annibale, Gianluigi Marseglia, Antonio Di Sabatino

**Affiliations:** ^1^ Department of Internal Medicine and Medical Therapeutics University of Pavia Pavia Italy; ^2^ First Department of Internal Medicine Fondazione IRCCS Policlinico San Matteo Pavia Italy; ^3^ Department of Medical‐Surgical Sciences and Translational Medicine, Sant'Andrea Hospital Sapienza University of Rome Rome Italy; ^4^ Vita‐Salute San Raffaele University Milan Italy; ^5^ Istituti Ospedalieri Bergamaschi Bergamo Italy; ^6^ Pediatric Unit, Department of Clinical, Surgical, Diagnostic and Pediatric Sciences University of Pavia Pavia Italy; ^7^ Pediatric Clinic Fondazione IRCCS Policlinico San Matteo Pavia Italy; ^8^ Pediatric Department Institute for Maternal and Child Health IRCCS “Burlo Garofolo” Trieste Italy; ^9^ Pediatric Gastroenterology and Endoscopy Unit IRCCS Istituto Giannina Gaslini Genova Italy; ^10^ Department of Neuroscience, Rehabilitation, Ophthalmology, Genetics, Child and Maternal Health University of Genoa Genoa Italy; ^11^ UOC Pediatria e neonatologia Gaslini Imperia Imperia Italy; ^12^ Interdisciplinary Department of Medicine, Pediatric Section, Children's Hospital ‘Giovanni XXIII’ University of Bari “Aldo Moro” Bari Italy; ^13^ Gastroenterology and Nutrition Unit Meyer Children's Hospital, IRCCS Florence Italy; ^14^ Department of NEUROFARBA University of Florence Florence Italy; ^15^ Pediatric Gastroenterology Unit Maggiore “CA Pizzardi” Hospital Bologna Italy; ^16^ Department of Pediatrics Fondazione IRCCS San Gerardo dei Tintori Monza Italy; ^17^ Institute of Pathology ASST‐Spedali Civili University of Brescia Brescia Italy; ^18^ Pediatric Gastroenterology and GI Endoscopy Unit, Children's Hospital ASST Spedali Civili University of Brescia Brescia Italy; ^19^ Pediatric Gastroenterology and Liver Unit, Maternal and Child Health Department University Hospital – Umberto I Sapienza University of Rome Rome Italy; ^20^ Pediatric Gastroenterology and Liver Unit Umberto I Hospital Rome Italy; ^21^ Pediatric Gastroenterology, Hepatology and Cystic Fibrosis Unit, Department of Pathophysiology and Transplantation Università degli Studi di Milano, Fondazione IRCCS Cà Granda, Ospedale Maggiore Policlinico di Milano Milan Italy; ^22^ The Juliet Keidan Institute of Pediatric Gastroenterology and Nutrition, Shaare Zedek Medical Center Jerusalem Israel; ^23^ The Faculty of Medicine The Hebrew University of Jerusalem Jerusalem; ^24^ Digestive Endoscopy and Gastroenterology Unit ASST Lecco Lecco Italy; ^25^ Pediatric Gastroenterology and Cystic Fibrosis Unit, Department of Human Pathology in Adulthood and Childhood “G. Barresi” University of Messina Messina Italy; ^26^ Department of Translational Medical Science Section of Pediatrics, University of Naples “Federico II” Naples Italy; ^27^ Department of Women, Child and General and Specialist Surgery University of Campania “Luigi Vanvitelli” Naples Italy; ^28^ Department of Surgery, Oncology, and Gastroenterology University of Padua Padua Italy; ^29^ Gastroenterology Unit Azienda Ospedale Università di Padova Padua Italy; ^30^ Unit of Pediatric Gastroenterology, Digestive Endoscopy, Hepatology and Care of the Child with Liver Transplantation, Department for Women's and Children's Health University Hospital of Padova Padova Italy; ^31^ Division of Pediatrics, Department of Mother and Child Health ASST Grande Ospedale Metropolitano Niguarda Milan Italy; ^32^ Pediatric Unit, Department of Neurosciences, Mental Health and Sensory Organs (NESMOS), Faculty of Medicine and Psychology Sapienza University of Rome, Sant'Andrea University Hospital Rome Italy; ^33^ Unit of Anatomic Pathology, Department of Molecular Medicine University of Pavia Pavia Italy; ^34^ Unit of Anatomic Pathology Fondazione IRCCS San Matteo Hospital Pavia Italy

**Keywords:** anemia, gastric atrophy, *Helicobacter pylori*, neuroendocrine neoplasm, vitamin B12

## Abstract

**Objectives:**

Autoimmune gastritis (AIG) has been poorly described in childhood. We sought to identify the patterns of manifestations of pediatric AIG at onset and to describe its laboratory, clinical, and histopathological features.

**Methods:**

This was a retrospective, longitudinal, multicenter, cohort study enrolling histologically proven AIG patients with an onset in the pediatric age (<18 years old). We retrieved laboratory and clinical data at the time of onset and at last follow‐up when available. Differences between *Helicobacter pylori*‐exposed versus *H. pylori*‐naïve, and anti‐parietal cell antibody (PCA)‐positive versus PCA‐negative patients were investigated.

**Results:**

Overall, 51 pediatric AIG patients (median age: 13 years, interquartile range: 11–16; F:M ratio 1.7:1) were included. Most patients were diagnosed with the overt type of AIG (47; 92.1%), while four (7.8%) were still in the potential phase. Atopic dermatitis (9.8%), rhinitis (7.8%), and asthma (5.9%) were common comorbidities, suggesting a link with T helper 2 (Th2) disorders. Two patients (3.9%) were found to have had previous or concurrent eosinophilic esophagitis, and five (9.8%) had eosinophilic gastritis. Notably, four patients (7.8%) presented with collagenous gastritis. On histological examination, the majority of patients were negative for *H. pylori* infection, except for 1 case out of 51 (2.0%) who had an active infection.

**Conclusions:**

AIG may affect pediatric patients and lead to complications in this population. At presentation, the disease may exhibit histologic patterns attributed to collagenous and/or eosinophilic gastritis. Moreover, a possible association between AIG and Th2 disorders has been observed, warranting further research.

## Abstract (highlights)

**What is Known:**

- Autoimmune gastritis (AIG) has been mostly described in adult patients.
- The clinical features of pediatric AIG are poorly understood.

**What is New:**

- In this retrospective, international, multicenter cohort study that included 51 pediatric AIG patients, T helper 2 disorders were frequently observed.
- In four cases, collagenous gastritis preceded AIG, and most patients were negative for *Helicobacter pylori*.
- Gastric neoplastic complications were rare.

## Graphical abstract

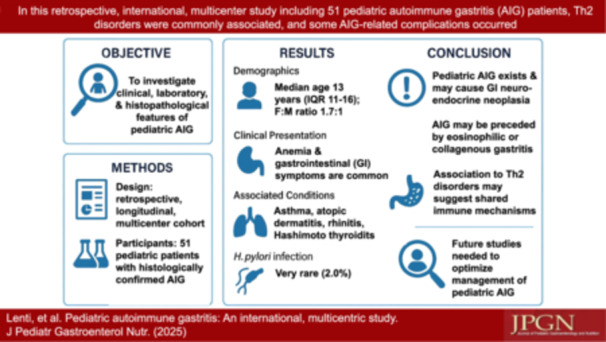

## 1 INTRODUCTION

Autoimmune gastritis (AIG) is a chronic inflammatory condition where the oxyntic mucosa of the stomach is affected by the immune activity against H^+^/K^+^‐ATPase in the parietal cells, leading to mucosal atrophy and vitamin B12, iron, and other micronutrient deficiency^‎^.^1^, ^2^, ^3^, ^4^ AIG has been described in individuals of all ages and genders, with a predominance in middle‐aged and elderly women.^‎^ ^1^, ^5^ Additionally, there is a strong link between AIG and other autoimmune comorbidities,^6^ and a first‐degree family history of AIG has been described in a substantial proportion of AIG patients,^7^ underlining the role of genetics in the development of this condition. Some environmental factors have also been reported, especially *Helicobacter pylori* (*H. pylori*) infection,^8^ though data are conflicting in adult AIG.‎

Conversely, AIG is considered to be very rare in the pediatric population or may be underdiagnosed. Actually, only a few studies have reported small case series of AIG in the pediatric age (Table S1).^9^, ^10^, ^11^, ^12^, ^13^, ^14^ Diagnosis in children is challenging due to its rarity and overlapping symptoms with other gastrointestinal conditions.‎ AIG patients may be asymptomatic or may have dyspeptic symptoms or iron deficiency anemia, which are also common in other pediatric conditions, such as celiac disease^‎^.^15^, ^16^ AIG may therefore be underestimated in the diagnostic workup, delaying the diagnosis and exposing the child to poor outcomes, as noticed in adult patients. In the long term, AIG may also lead to complications, including gastric neuroendocrine neoplasms^17^, ^18^ and gastric adenocarcinoma.^1^ Hence, a timely diagnosis of AIG in children is of utmost importance. Currently, the long‐term natural history of pediatric‐onset AIG is unknown, and the clinical and pathological features have been poorly described.

In this multicenter study, we have attempted to identify the patterns of presentation and clinical features of pediatric AIG, to facilitate prompt recognition and, possibly, diagnosis at early stages.

## 2 METHODS

### 2.1 Ethics statement

Written informed consent was provided by all patients and/or their parents or caregivers for the anonymous publication of their data. All data have been completely anonymized and aggregated. The study was approved by the local Ethics Committee (Fondazione IRCCS Policlinico San Matteo, protocol number 0003262/25). Each center was responsible for the approval of collecting clinical data at their single Units. All data produced in the present research are reported in this paper. Additional raw data can be shared upon reasonable request to the corresponding author. Patients or members of the public were not involved in the design, conduct, reporting, or dissemination plans of the present research.

### 2.2 Study population and inclusion criteria

This multicenter retrospective study included participants below 18 years of age at the time of diagnosis, and diagnosed between 2019 and 2024 with any type and stage of AIG, including potential,^19^ overt or seronegative AIG.^20^ Potential AIG was defined as the presence of anti‐parietal cell antibody (PCA) in patients without histological evidence of oxyntic mucosal atrophy. These patients should be closely monitored, as they may progress to overt AIG over time, as demonstrated in the adult population.^19^ Overt AIG refers to cases in which any degree of atrophy (ranging from mild to severe) is present in the oxyntic mucosa.^1^ Seronegative AIG is defined as histologically confirmed atrophy of the oxyntic mucosa in the absence of detectable circulating PCA.^20^ Pediatric participants were recruited from 14 centers, including 13 from Italy and one from Israel, based on the local expertise in the diagnosis of gastroenterological diseases in children and adolescents. The participants recruited were either patients tested for PCA for any reason, such as screening purposes, family history, dyspeptic symptoms, anemia, or other symptoms that raised the suspicion of AIG, or patients who directly received a diagnosis of AIG by upper gastrointestinal endoscopy with biopsies (e.g., also including seronegative AIG patients). AIG diagnosis was always made on a histological basis in those that demonstrated any degree of atrophy in the oxyntic gastric mucosa, with antrum sparing, according to internationally recognized criteria.^21^ In all cases, the Sydney–Houston protocol^22^ for collecting biopsies was used, that is, two biopsies from the antrum, one from the incisura angularis, two from the corpus. The mean overall time of observation from the time of diagnosis to the last follow‐up was calculated.

### 2.3 Timepoints and variables collected

Sociodemographic (age, sex), clinical (symptoms at presentation), laboratory (vitamin B12, folic acid, ferritin, gastrin‐17, and chromogranin A), and endoscopic data (at the time of diagnosis and last follow‐up, when available) were collected. *H. pylori* status was considered, along with previous eradication therapies; other comorbidities, including autoimmune and non‐autoimmune ones, family history for autoimmunity, AIG, and malignancies, immunodeficiencies, prior proton pump inhibitor (PPI) use, and other pharmacologic therapies were among other variables identified.

### 2.4 Study end points

The primary objective of the study was to report the characteristics of AIG pediatric patients. We also reported the occurrence of gastric neuroendocrine neoplasms as well as dysplasia or adenocarcinoma during the whole time of observation. As a secondary objective, we assessed patients' characteristics in relation to the age groups ≥12 or <12 years and to the serological status (PCA positivity or negativity).

### 2.5 Statistical analysis

The software MedCalc (online, free version) was used for all computations. A two‐sided *p* < 0.05 was considered statistically significant. Continuous data were described either with median and interquartile range (IQR; i.e., 25th–75th percentiles) or mean and standard deviation (SD), while categorical data were presented as counts and percentages. A Fisher's exact test was performed for comparative purposes, for example, for comparing seropositive versus seronegative patients, and for comparing different age groups. The cumulative time of observation was calculated. The STrengthening the Reporting of OBservational studies in Epidemiology guidelines were followed for quality assurance.

## 3 RESULTS

Overall, 51 pediatric AIG patients (median age at diagnosis: 13 years, IQR: 11–16; F:M ratio: 1.7:1) were included in the study (Table 1). The median time of observation was 24 months (IQR: 12–57). AIG was more common in female patients and among individuals 12 years or older. There were no active smokers, and most of the patients included were Caucasian.

**Table 1:** Main demographic characteristics of 51 pediatric patients with autoimmune gastritis.

Age at diagnosis (years), median (IQR)	13 (11–16)
Age groups, years, *n* (%)	
≥12	34 (66.7)
<12	17 (33.3)
Sex, *n* (%)	
Male	19 (37.2)
Female	32 (62.7)
Ethnicity, *n* (%)	
Asian	0 (0)
African	3 (6.2)
Hispanic/Latino	0 (0)
White/Caucasian	44 (91.7)
Arab	1 (2.1)
Jewish	3 (6.2)
Breastfeeding, *n* (%)	
No	17 (33.3)
Yes	16 (31.4)
Partial	13 (25.5)
Unknown	5 (9.8)
Birth modality, *n* (%)	
Vaginal birth	31 (25.5)
Cesarean section	11 (21.6)
Unknown	9 (17.6)

Abbreviation: IQR, interquartile range.

The majority of patients were diagnosed with overt AIG (47; 92.2%), while four (7.8%) were still in the potential phase (Table 2). Regarding hematologic manifestations at diagnosis, the most common was microcytosis (33; 64.7%), while three (5.9%) had macrocytosis, and two patients (3.9%) had pancytopenia. Overt anemia was present in most cases (38; 74.5%), with the mild form being most common (22; 43.1%). Immunoglobulin A deficiency was present in two participants (3.9%), while one patient (2.0%) was affected by common variable immunodeficiency, and four (7.8%) had concomitant polyglandular syndrome. Atopic dermatitis, rhinitis, and asthma were present in five (9.8%), four (7.8%), and three (5.9%) patients, respectively. *H. pylori* infection was very rare (1; 2.0%), and one had a previously eradicated infection (2.0%). PCA positivity was present in 38 (74.5%) patients.

**Table 2:** Clinical characteristics of the 51 pediatric patients with autoimmune gastritis.

Autoimmune gastritis, *n* (%)	
Potential	4 (7.8)
Overt	47 (92.1)
Hematologic manifestations, *n* (%)	
None	13 (25.5)
Microcytosis	33 (64.7)
Macrocytosis	3 (5.9)
Pancytopenia	2 (3.9)
Anemia, *n* (%)	
No anemia	13 (25.5)
Mild anemia	22 (43.1)
Moderate anemia	12 (23.5)
Severe anemia	4 (7.8)
Hyperhomocysteinemia, *n* (%)	2 (3.9)
Gastroenterological manifestations, *n* (%)	
None	23 (45.1)
Dyspepsia	13 (25.5)
Gastroesophageal reflux disease	5 (9.8)
Diarrhea	3 (5.9)
Abdominal pain	13 (25.5)
Weight loss	2 (3.9)
Neurological manifestations, *n* (%)	4 (7.8)
Type of neurologic manifestations, *n* (%)	
Paresthesia	1 (2)
Psychiatric condition	3 (5.9)
Memory loss	0 (0)
Other	1 (2)
Concomitant comorbidities, *n* (%)	
None	43 (84.3)
Obesity	2 (3.9)
Hypertension	1 (2)
Liver cirrhosis	1 (2)
Familiar polyposis	1 (2)
Hyperlipidemia	1 (2)
Esophageal stenosis	1 (2)
Nonceliac villous atrophy	1 (2)
Kabuki syndrome	1 (2)
Seizures	1 (2)
Immunodeficiency, *n* (%)	
CVID	1 (2)
STAT 1 deficiency	1 (2)
IgA deficiency	2 (3.9)
Hypogammaglobulinemia	1 (2)
Family history for gastric neoplasia, *n* (%)	
No	39 (76.5)
Yes	2 (3.9)
Unknown	2 (3.9)
Concomitant autoimmune polyglandular syndrome, *n* (%)	
No	46 (90.2)
Yes	4 (7.8)
Unknown	1 (2)
Concomitant atopy, *n* (%)	
None	40 (78.4)
Allergic rhinitis	4 (7.8)
Atopic dermatitis	5 (9.8)
Asthma	3 (5.9)
Prior PPI therapy for >3 months, *n* (%)	12 (23.5)
*H. pylori* status, *n* (%)	
Negative	46 (90.2)
Positive	1 (2)
Eradicated	1 (2)
Unknown	3 (5.9)
PCA status, *n* (%)	
Negative	8 (15.7)
Positive	38 (74.5)
Unknown/not available	5 (9.8)

Abbreviations: CVID, common variable immune deficiency; *H. pylori*, *Helicobacter pylori*; IgA, immunoglobulin A; PCA, parietal cell antibody; PPI, proton pump inhibitor; STAT1, signal transducer and activator of transcription 1.

Four patients (7.8%) had a family history of AIG, and 17 (33.3%) had a family history of autoimmune diseases in general (Table S2). Thirty‐five patients (68.6%), however, had a concomitant autoimmune disorder, with the most common being Hashimoto's thyroiditis (19/51; 37.2%), followed by celiac disease (5/51; 9.8%), type 1 diabetes, and vitiligo each affecting three patients (5.9%). Two patients (3.9%) were found to have had previous or concurrent eosinophilic esophagitis, and five (9.8%) had eosinophilic gastritis. Table S3 reports the relevant baseline and last available laboratory data of patients with AIG.

Concerning the characteristics of patients according to the age groups <12 and ≥12 years (Table S4), patients in the older group were more likely to receive a diagnosis of neuroendocrine neoplasm earlier compared to the younger group (median 12 months vs. 84 months after AIG diagnosis, *p* = 0.003). No other significant differences were noticed. Table S5 reports the laboratory data based on the age groups.

Regarding differences between PCA‐positive and PCA‐negative (i.e., seronegative) AIG patients (Table S6), pancytopenia was significantly more common in the PCA‐negative group (2/8; 25% vs. 0; 0%; *p* = 0.0018). Also, microcytosis was more common in the PCA‐negative group (25/38; 65.8%), compared to 1/8 of PCA‐positive patients (12.5%; *p* = 0.0063). Dyspepsia was the most common gastrointestinal manifestation in the PCA‐positive patients (12/38; 31.6%), while no one in the PCA‐negative group presented with the symptom. A misdiagnosis of functional dyspepsia and irritable bowel syndrome was also more common in the PCA negative group (*p* = 0.0255 and 0.0293, respectively). PCA‐negative patients were also more likely to receive a diagnosis of neuroendocrine neoplasm earlier than PCA‐positive patients (median 12 months vs. 84 months; *p* = 0.002). No other significant differences were noticed. Table S7 reports the laboratory data based on PCA status.

On histological examination, the majority of patients were negative for *H. pylori* infection, except 1 case out of 51 (2.0%) who had an active infection, subsequently eradicated (Table 3). Consistent with a diagnosis of AIG, corpus mucosal atrophy was present in most patients, except for the four potential AIG patients, while intestinal metaplasia was seen in 11 out of 51 (21.6%) of the cases. Notably, four patients (7.8%) had collagenous gastritis with prominent eosinophilia as presenting manifestation, which then evolved in all cases into overt AIG within 2 years.

**Table 3:** Endoscopic and histopathological findings of the corpus/fundus mucosa of the 51 pediatric patients with autoimmune gastritis.

Macroscopic findings, *n* (%)	
None	18 (35.3)
Flattened mucosa	14 (27.4)
Pale mucosa	3 (5.9)
Hyperemic mucosa	14 (27.4)
Areas suspicious for metaplasia	2 (3.9)
Polyps	1 (2)
Early lesions, *n* (%)	
None	23 (45.1)
Mononuclear cells infiltration without loss of native glands	18 (35.3)
*H. pylori* infection, *n* (%)	1 (2.0)
Severity of mucosal atrophy, *n* (%)	
Absent	4 (7.8)
Mild	18 (35.3)
Moderate	10 (19.6)
Severe	13 (25.5)
Severity not specified	6 (11.8)
Intestinal metaplasia, *n* (%)	11 (21.6)
Complete intestinal metaplasia, *n* (%)	
Absent	31 (60.8)
Mild	8 (15.7)
Moderate	1 (2)
Severe	0 (0)
Unknown	11 (21.6)
Incomplete intestinal metaplasia, *n* (%)	
Absent	4 (66.7)
Mild	6 (11.8)
Moderate	1 (2)
Severe	0 (0)
Unknown	10 (19.6)
Pseudopyloric metaplasia, *n* (%)	
Absent	34 (66.7)
Mild	10 (19.6)
Moderate	0 (0)
Severe	1 (2)
Unknown	6 (11.8)
ECL cell hyperplasia, *n* (%)	
No	15 (29.4)
Yes	24 (47)
Unknown	12 (23.52)
NET, *n* (%)	7 (13.7)
Epithelial dysplasia, *n* (%)	7 (13.7)

Abbreviations: ECL, enterochromaffin‐like; *H. pylori*, *Helicobacter pylori*; NET, neuroendocrine tumor.

**Figure 1:**
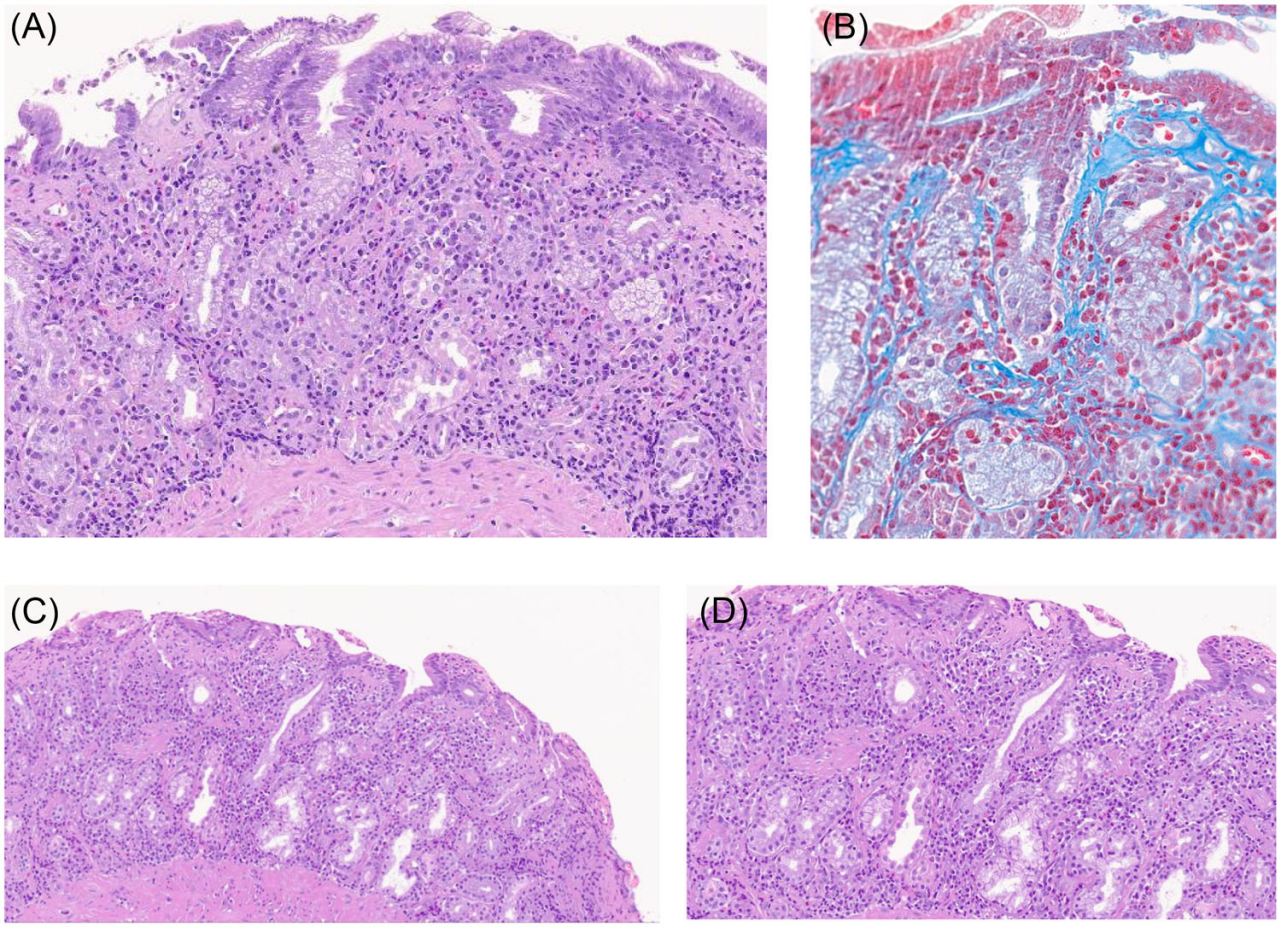
Histopathological specimen of the gastric corpus of a patient who was diagnosed with collagenous gastritis (A, B) and subsequently evolved into autoimmune gastritis (C, D) after roughly 2 years. In (A), the hematoxylin and eosin staining shows increased eosinophil infiltration of the mucosa with a thickening of the subepithelial collagen, better noticed with the Masson‐trichrome staining (B); a very mild glandular atrophy can be noticed. In (C and D), after 2 years, typical features of autoimmune gastritis are noticed with hematoxylin and eosin staining, namely mucosal atrophy; the subepithelial collagen band is not clearly evident anymore.

Figure 1 shows a representative histopathological specimen of a patient who was diagnosed with collagenous gastritis (A, B), and subsequently evolved into AIG (C, D). The occurrence of neuroendocrine neoplasms and epithelial glandular dysplasia (low‐grade in all cases) was 7 out of 51 (13.7%) each, while none of the patients had overt gastric adenocarcinoma during the observation period.

## 4 DISCUSSION

This multicenter study reports on 51 pediatric AIG patients from 14 centers in Italy and Israel, contributing to the growing body of knowledge of this condition. Unlike prior studies, our longitudinal approach offers valuable insights into the natural history and clinical evolution of AIG in children and adolescents. We included patients with various stages and subtypes of AIG, ranging from potential to overt and from seropositive to seronegative. The patients were clinically followed throughout 24 months (median, IQR: 12–57), which is usually a lacking factor in the previous literature.

In this study, we have provided evidence regarding various characteristics of AIG in the pediatric population. These new findings include the possible correlation of pediatric AIG with Th2‐related disorders such as allergic rhinitis and asthma; the scarcity of *H. pylori* infection in children affected by AIG; a link with collagenous gastritis at the very beginning of the disease; a common encounter of seronegative AIG in children and a high frequency of immune‐mediated diseases and immunodeficiencies in these individuals.

As mentioned, *H. pylori* infection was a rare occurrence in our study population. Previously, on a study on *H. pylori*‐associated and autoimmune‐derived gastritis, we demonstrated that in adults atrophic gastritis can result from either autoimmune reactivity of the immune cells and antibodies against the gastric oxyntic mucosa, or the result of a progressed *H. pylori* infection.^7^ In children, however, it seems that *H. pylori* infection is very rare (1/51, 2%) and that autoimmunity plays a more important role than infectious origins in the development of atrophic gastritis.

In the context of AIG, PCA, antibodies against the H^+^/K^+^ ATPase component of the gastric parietal cells are the marker of disease and are used for screening‎.^23^ Nevertheless, we have found that in children affected by AIG, PCA‐negativity is rather common. This unexpected high incidence of seronegative AIG in pediatric patients demands attentive diagnostic strategies. So far, PCA negativity had been linked to AIG in the elderly, possibly to an immuno‐senescence mechanism.^24^

In this study, seronegative AIG patients tend to get misdiagnosed with other disorders, such as a different type of anemia, irritable bowel syndrome, leukemia, and, more importantly, different types of gastritis. This is particularly relevant given that PPIs are a mainstay of therapy for various types of gastritis, with the exception of AIG‎.^25^ In AIG, PPIs are contraindicated as they may facilitate the progression of the disease‎ into neuroendocrine neoplasms.^26^ Children, having a longer life expectancy, may be more prone to long‐term complications of AIG, namely adenocarcinoma or gastric neuroendocrine neoplasms; a misdiagnosis and mismanagement of the condition can, therefore, further expose the patient to adverse disease outcomes^‎^.^13^, ^27^

Our study also provides some possible pathophysiological insights into AIG in the pediatric population. AIG is correlated with an initial appearance of eosinophilic infiltration and sub‐epithelial thickening of the oxyntic mucosa, consistent with a diagnosis of collagenous gastritis before the occurrence of any other more typical pathological signs of AIG^‎^.^28^, ^29^ Eosinophils may play a significant role in the immunomodulation, steering the immune system towards autoreactivity and production of antibodies against the lining cells of the oxyntic mucosa. Consistently, in adult AIG, the oxyntic mucosa usually displays eosinophilic infiltration^‎^.^1^, ^30^ The high occurrence of Th2‐related conditions (e.g., asthma, atopy, and eosinophilic esophagitis) and other autoimmune diseases (e.g., Hashimoto's thyroiditis and celiac disease) suggests a shared immunological pathway. Furthermore, eosinophilic gastritis and esophagitis were notably common in our study cohort. This finding highlights a possible immunomodulatory role of eosinophils in driving autoimmunity and mucosal atrophy, warranting further exploration into the interplay between eosinophils and AIG pathogenesis.

The natural history of AIG progression from potential to overt stages remains poorly understood in children. Our study revealed that some patients initially diagnosed with collagenous gastritis transitioned to overt AIG within 2 years, underscoring the need for ongoing monitoring of at‐risk children. More extended studies are required to address longer follow‐up and monitoring of disease progression to obtain a deeper insight into the advancement of AIG in children.

Neuroendocrine neoplasms and epithelial dysplasia were observed in 13.7% of our cohort, emphasizing the importance of early diagnosis. Mismanagement due to delayed diagnosis may increase the risk of adverse outcomes, including gastric adenocarcinoma, particularly given the longer life expectancy of pediatric patients.

Indeed, our study has many limitations that should be mentioned. Despite this being one of the largest studies published so far in pediatric AIG, the sample size is relatively small and does not allow for a more granular analysis. The retrospective nature has intrinsic limitations, such as selection bias, potential confounding factors, and data heterogeneity. Also, compared to adult AIG studies, longer follow‐up is needed to better highlight the risk of developing complications. Finally, a larger, prospective study also including other countries and diverse ethnicities is warranted.

## 5 CONCLUSION

To conclude, AIG may affect pediatric patients and may also lead to complications in this population. A certain association with Th2 disorders was noticed, and this finding warrants further research attention. Further research is needed to elucidate the mechanisms underlying these associations and to optimize management strategies for pediatric patients.

## CONFLICT OF INTEREST STATEMENT

The authors declare no conflicts of interest.

## Supporting information

Table S1. 08May25.docx.

Table S2. 08May25.docx.

Table S3. 08May25.docx.

Table S4. 08May25.docx.

Table S5. 08May25.docx.

Table S6. 08May25.docx.

Table S7. 08May25.docx.
