# Supplementary material for: Pediatric autoimmune gastritis: An international, multicentric study
Source: J Pediatr Gastroenterol Nutr. 2025 Aug 12;81(5):1142–50. doi: 10.1002/jpn3.70187 (PMC12580456; doi:10.1002/jpn3.70187)
Supplement: Supplementary file 1 — Table S1. 08May25.docx. [file JPN3-81-1142-s001.docx]

**Supplementary Table 1.** Summary of the main studies or case series reporting on pediatric autoimmune gastritis (AIG).

| **First author, year** | **Country** | **Design** | **# patients with AIG** | **Main findings** |
| --- | --- | --- | --- | --- |
| Segni, 2004 | Italy | Cohort study, prospective | 5 | Of 129 screened children with autoimmune thyroid disease, 30% had positive anti-parietal cell antibodies, and five patients a histologically confirmed AIG |
| Gonçalves, 2014 | Portugal | Case series, retrospective | 5 | Iron deficiency anemia and family history for autoimmunity are very common in pediatric AIG |
| Saglietti, 2018 | Switzerland | Case series, retrospective | 2 | Iron deficiency anemia as presenting manifestation in both; no family history of autoimmunity |
| Mitsinikos, 2020 | United States | Case series, retrospective | 3 | Pernicious anemia, other types of anemia and autoimmune comorbidities/history should raise suspicion of AIG |
| Kulak, 2021 | United States | Case series, retrospective | 22 | Extra-gastric immune disorders should be considered when a diagnosis of pediatric AIG is established |
| Granot, 2024 | United States | Multicenter, retrospective | 33 | AIG should be considered in patients with autoimmunity and resistant iron‐deficiency anemia; *H. pylori* infection may not be associated with pediatric AIG; one patient had a neuroendocrine neoplasm. |
