# Supplementary material for: Pediatric autoimmune gastritis: An international, multicentric study
Source: J Pediatr Gastroenterol Nutr. 2025 Aug 12;81(5):1142–50. doi: 10.1002/jpn3.70187 (PMC12580456; doi:10.1002/jpn3.70187)
Supplement: Supplementary file 2 — Table S2. 08May25.docx. [file JPN3-81-1142-s005.docx]

**Supplementary Table 2.** Prevalence of autoimmune and immune mediated diseases in the 51 pediatric patients with autoimmune gastritis (AIG).

| Family history for AIG, n (%) | 4 (7.8) |
| --- | --- |
| Family history for autoimmunity, n (%)  None  Yes  Unknown | 35 (68.6)  17 (33.3)  3 (5.9) |
| Associated autoimmune disorder, n (%) | 35 (68.6) |
| Type of associated autoimmune and immune-mediated disorders, n (%)  None  Hashimoto’s thyroiditis  Grave’s disease  Vitiligo  Diabetes mellitus type I  Celiac disease  Posterior uveitis  Alopecia areata  Autoimmune hepatitis  Atopic dermatitis  Ulcerative colitis  Autoimmune hemolytic syndrome  Crohn’s disease  IgA deficiency | 20 (39.2)  19 (37.2)  1 (2.1)  3 (5.9)  3 (5.9)  5 (9.8)  1 (2)  1 (2)  1 (2)  1 (2)  1 (2)  1 (2)  1 (2)  1 (2) |
| Total number of autoimmune comorbidities, n (%)  0  1  2  3 | 25 (49)  19 (37.2)  6 (11.8)  1 (2) |
| Concomitant autoimmune polyglandular syndrome, n (%)  None  Yes  Suspected | 46 (90.2)  4 (7.8)  1 (2) |
| Concomitant gastrointestinal eosinophilic disorder, n (%)  None  Esophagitis  Gastritis  Unknown | 43 (84.3)  2 (3.9)  5 (9.8)  1 (2) |
