# Supplementary material for: Pediatric autoimmune gastritis: An international, multicentric study
Source: J Pediatr Gastroenterol Nutr. 2025 Aug 12;81(5):1142–50. doi: 10.1002/jpn3.70187 (PMC12580456; doi:10.1002/jpn3.70187)
Supplement: Supplementary file 3 — Table S3. 08May25.docx. [file JPN3-81-1142-s004.docx]

**Supplementary Table 3**. Baseline and last available laboratory data of patients with autoimmune gastritis.

| Variable | Mean (SD), baseline | Mean (SD), last available |
| --- | --- | --- |
| Vitamin B12, pg/ml | 353.7 (152.3) | NA |
| Folic acid, ng/ml | 7.3 (9.5) | NA |
| Ferritin, ng/ml | 13.6 (21.3) | NA |
| Gastrin 17, pg/ml | 575.4 (241.0) | 642.5 (301.2) |
| Chromogranin A, ng/ml | 92.6 (29.3) | 83.13 (35.2) |

Abbreviation: SD, standard deviation.
