# Supplementary material for: Pediatric autoimmune gastritis: An international, multicentric study
Source: J Pediatr Gastroenterol Nutr. 2025 Aug 12;81(5):1142–50. doi: 10.1002/jpn3.70187 (PMC12580456; doi:10.1002/jpn3.70187)
Supplement: Supplementary file 4 — Table S4. 08May25.docx. [file JPN3-81-1142-s006.docx]

**Supplementary Table 4**. Prevalence of clinical characteristics in pediatric patients with autoimmune gastritis, based on the age group.

|  | **≥12 years old** | **<12 years old** | **p value** |
| --- | --- | --- | --- |
| Autoimmune gastritis, n (%)  Potential  Overt | 4 (11.8)  30 (88.2) | 1 (5.9)  16 (94.1) | 0.5089 |
| Hematologic manifestations, n (%)  None  Microcytosis  Macrocytosis  Pancytopenia | 9 (26.5)  20 (58.8)  3 (8.8)  2 (5.9) | 4 (23.5)  13(76.5)  0 (0)  0 (0) | 0.8185  0.2170  0.2119  0.3117 |
| Anemia severity, n (%)  No anemia  Mild anemia  Moderate anemia  Severe anemia | 10 (29.4)  12 (35.3)  9 (26.5)  3 (8.8) | 3 (17.6)  10 (58.8)  3 (17.6)  1 (5.9) | 0.3666  0.1137  0.4843  0.7190 |
| Gastroenterological symptoms, n (%) | 19 (55.9) | 9 (52.9) | 0.8460 |
| Gastroenterological manifestations, n (%)  Dyspepsia  Reflux  Diarrhea  Abdominal pain  Weight loss | 9 (26.5)  4 (11.8)  2 (5.9)  11 (32.3)  2 (5.9) | 4 (23.5)  1 (5.9)  1 (5.9)  2 (11.8)  0 (0) | 0.8185  0.5089  1.0000  0.1168  0.3117 |
| Neurological manifestation, n (%) | 4 (11.8) | 1 (5.9) | 0.5089 |
| Type of neurologic manifestations, n (%)  Paresthesia  Psychiatric condition  Memory loss  Other | 0 (0)  3 (8.8)  0 (0)  1 (2.9) | 1 (5.9)  0 (0)  0 (0)  0 (0) | 0.1567  0.2119    0.4827 |
| Total number of autoimmune comorbidities, n (%)  0  1  2  3 | 16 (47)  15 (44.1)  2 (5.9)  1 (2.9) | 9 (52.9)  4 (23.5)  4 (23.5)  0 (0) | 0.6940  0.1555  0.0686  0.4827 |
| Associated autoimmune disorder, n (%) | 24 (70.6) | 11 (64.7) | 0.6717 |
| Concomitant comorbidities, n (%)  Obesity  Hypertension  Liver cirrhosis  Collagenous gastritis  Esophageal stenosis  Non-celiac villous atrophy  Familiar polyposis | 2 (5.9)  1 (2.9)  1 (2.9)  1 (2.9)  1 (2.9)  1 (2.9)  0 (0) | 0 (0)  0 (0)  0 (0)  0 (0)  0 (0)  0 (0)  1 (5.9) | 0.3117  0.4827  0.4827  0.4827  0.4827  0.4827  0.1567 |
| Type of associated autoimmune and immune-mediated disorders, n (%)  None  Hashimoto’s thyroiditis  Grave’s disease  Vitiligo  Diabetes mellitus type I  Celiac disease  Posterior uveitis  Autoimmune hepatitis  Atopic dermatitis  Autoimmune hemolytic syndrome  Crohn’s disease  Ulcerative colitis  IgA deficiency  Alopecia areata | 13 (38.2)  12 (35.3)  1 (2.9)  2 (5.9)  2 (5.9)  2 (5.9)  1 (2.9)  1 (2.9)  1 (2.9)  1 (2.9)  1 (2.9)  0 (0)  1 (2.9)  0 (0) | 7 (41.2)  7 (41.2)  0 (0)  1 (5.9)  1 (5.9)  3 (17.6)  0 (0)  0 (0)  0 (0)  0 (0)  0 (0)  1 (5.9)  0 (0)  1 (5.9) | 0.8377  0.6842  0.4827  1.000  1.0000  0.1896  0.4827  0.4827  0.4827  0.4827  0.4827  0.1567  0.4827  0.1567 |
| Immunodeficiency, n (%)  CVID  STAT 1 deficiency  IgA deficiency  Hypogammaglobulinemia  LBRA deficiency | 0 (0)  1 (2.9)  2 (5.9)  0 (0)  0 (0) | 1 (5.9)  0 (0)  0 (0)  1 (5.9)  1 (5.9) | 0.1567  0.4827  0.3117  0.1567  0.1567 |
| Family history for AIG, n (%)  No  Yes  Unknown | 29 (85.3)  3 (8.8)  2 (5.9) | 16 (94.1)  1 (5.9)  0 (0) | 0.3626  0.7190  0.3117 |
| Family history for gastric neoplasia, n (%)  No  Yes  Unknown | 30 (88.2)  2 (5.9)  2 (5.9) | 16 (94.1)  1 (5.9)  0 (0) | 0.5089  1.0000  0.3117 |
| Family history for autoimmunity, n (%)  No  Yes  Unknown | 21(61.8)  10 (29.4)  3 (8.8) | 10 (58.8)  7 (41.2)  0 (0) | 0.8377  0.4041  0.2119 |
| Concomitant autoimmune polyglandular syndrome, n (%)  No  Yes  Suspected | 30 (88.2)  3 (11.8)  1 (2.9) | 16 (94.1)  1 (5.9)  0 (0) | 0.5089  0.5089  0.4827 |
| Prior PPI therapy for >3 months, n (%) | 8 (23.5) | 4 (23.5) | 1.0000 |
| *H. pylori* status, n (%)  Negative  Positive  Eradicated  Unknown | 31 (91.2)  1 (2.9)  1 (2.9)  1 (2.9) | 15 (87.5)  0 (0)  0 (0)  2 (11.8) | 0.6814  0.4827  0.4827  0.2068 |
| PCA status, n (%)  Negative  Positive  Unknown | 6 (17.6)  26 (76.5)  2 (5.9) | 2 (11.8)  12 (70.6)  3 (17.6) | 0.5948  0.6517  0.1896 |
| Factors leading to diagnosis, n (%)  Gastrointestinal impairment  Hematological impairment  Autoimmune screening  Family history  Immunodeficiency | 8 (23.5)  19 (55.9)  14 (41.2)  1 (2.9)  1 (2.9) | 5 (29.4)  8 (47)  3 (17.6)  1 (5.9)  0 (0) | 0.6517  0.5523  0.0952  0.6055  0.4827 |
| Previous misdiagnosis, n (%)  No  Yes  Unknown | 22 (64.7)  12 (35.3)  0 (0) | 11 (64.7)  4 (23.5)  1 (5.9) | 1.0000  0.3966  0.1567 |
| Misdiagnosis, n (%)  Dyspepsia-other gastritis  Gastroesophageal reflux disease  Other form of anemia  Leukemia | 4 (11.7)  1 (2.9)  6 (17.6)  1 (2.9) | 4 (23.5)  2 (11.8)  0 (0)  0 (0 | 0.2788  0.2068  0.0683  0.4827 |
| Complication at onset, n (%) | 14 (41.2) | 9 (52.9) | 0.2399 |
| Type of complication at onset, n (%)  Pernicious anemia  Iron deficiency anemia  Gastric NETs | 2 (5.9)  15 (44.1)  0 (0) | 0 (0)  10 (58.8)  1 (5.9) | 0.3117  0.3270  0.1567 |
| Gastric NET, n (%)  No  Yes  Unknown | 32 (94.1)  1 (2.9)  1 (2.9) | 15 (88.2)  1 (5.9)  1 (5.9) | 0.4651  0.6055  0.6055 |
| Hyperhomocysteinemia, n (%) | 1 (2.9) | 1 (5.9) | 0.6204 |
| Months from diagnosis to NET, median of months (IQR) | 12 (7-15) | 84 (68-101) | 0.003 |
| Total time of observation in months, mean (SD) | 37.6 (33.95) | 38.2 (33.74) | 0.9527 |
| Total diagnostic delay in months, mean (SD) | 12.2 (11.07) | 11.5 (9.81) | 0.8262 |

Abbreviations: CVID, common variable immune deficiency; IQR, interquartile range; NET, neuroendocrine tumor; PCA, anti-parietal cell antibodies; PPI, proton pump inhibitor; SD, standard deviation.
