# Supplementary material for: Pediatric autoimmune gastritis: An international, multicentric study
Source: J Pediatr Gastroenterol Nutr. 2025 Aug 12;81(5):1142–50. doi: 10.1002/jpn3.70187 (PMC12580456; doi:10.1002/jpn3.70187)
Supplement: Supplementary file 5 — Table S5. 08May25.docx. [file JPN3-81-1142-s002.docx]

**Supplementary Table 5**. Laboratory data based on age groups.

|  | **≥12 years old** | **<12 years old** | **p value** |
| --- | --- | --- | --- |
| Vitamin B12 level at onset, mean (SD) | 351.7 (210.89) | 358.8 (208.56) | 0.9099 |
| Folic acid level at onset, mean (SD) | 7.41 (5.45) | 7.40 (5.58) | 0.9951 |
| Ferritin level at onset, mean (SD) | 13.9 (13.97) | 13.7 (14.06) | 0.9618 |
| Gastrin-17 level at onset, mean (SD) | 592.9 (960) | 575.4 (962.2) | 0.9514 |
| Chromogranin level at onset, mean (SD) | 70.4 (170.23) | 98.9 (168.63) | 0.5744 |
| Last available gastrin-17 level, mean (SD) | 656.1 (841.65) | 642.5 (792.75) | 0.9560 |
| Last available chromogranin level, mean (SD) | 84.4 (144.8) | 83.8 (145.11) | 0.9889 |

Abbreviation: SD, standard deviation.
