# Supplementary material for: Pediatric autoimmune gastritis: An international, multicentric study
Source: J Pediatr Gastroenterol Nutr. 2025 Aug 12;81(5):1142–50. doi: 10.1002/jpn3.70187 (PMC12580456; doi:10.1002/jpn3.70187)
Supplement: Supplementary file 6 — Table S6. 08May25.docx. [file JPN3-81-1142-s003.docx]

**Supplementary Table 6**. Clinical data based on anti-parietal cell antibody (PCA) status.

|  | **PCA positive** | **PCA negative** | **p value** |
| --- | --- | --- | --- |
| Autoimmune gastritis, n (%)  Potential  Overt | 3 (7.9)  35 (92.1) | 1 (12.5)  7 (87.5) | 0.6781 |
| Hematologic manifestations, n (%)  None  Microcytosis  Macrocytosis  Pancytopenia | 11 (28.9)  25 (65.8)  2 (5.3)  0 (0) | 1 (12.5)  1 (12.5)  1 (12.5)  2 (25) | 0.3421  0.0063  0.4594  0.0018 |
| Anemia severity, n (%)  No anemia  Mild anemia  Moderate anemia  Severe anemia | 11 (28.9)  17 (44.7)  8 (21)  2 (5.3) | 1 (12.5)  3 (37.5)  2 (25)  2 (25) | 0.3421  0.7119  0.8051  0.0759 |
| Gastroenterological symptoms, n (%) | 21 (55.3) | 5 (62.5) | 0.7119 |
| Gastroenterological manifestations, n (%)  None  Dyspepsia  Gastroesophageal reflux disease  Diarrhea  Abdominal pain  Weight loss | 19 (50)  12 (31.6)  4 (10.5)  0 (0)  9 (23.7)  1 (2.6) | 3 (37.5)  0 (0)  1 (12.5)  3 (37.5)  4 (50)  1 (12.5) | 0.5246  0.0673  0.8701  0.0001  0.1376  0.2158 |
| Neurological manifestations, n (%) | 3 (7.9) | 1 (12.5) | 0.6781 |
| Type of neurologic manifestations, n (%)  Psychiatric condition  Other | 2 (5.3)  1 (2.6) | 1 (12.5)  0 (0) | 0.4594  0.6484 |
| Total number of autoimmune comorbidities, n (%)  0  1  2  3 | 15 (39.5)  16 (42.1)  6 (15.8)  1 (2.6) | 5 (62.5)  3 (37.5)  0 (0)  0 (0) | 0.2381  0.8122  0.2331  0.6484 |
| Associated autoimmune disorder, n (%) | 28 (73.7) | 5 (62.5) | 0.5270 |
| Concomitant comorbidities, n (%)  Obesity  Hypertension  Liver cirrhosis  Kabuki syndrome  Chronic pancreatitis | 2 (5.3)  1 (2.6)  1 (2.6)  0 (0)  0 (0) | 0 (0)  0 (0)  0 (0)  1 (12.5)  1 (12.5) | 0.5101  0.6484  0.6484  0.0293  0.0293 |
| Type of associated autoimmune and immune-mediated disorders, n (%)  None  Hashimoto’s thyroiditis  Grave’s disease  Vitiligo  Diabetes mellitus type I  Coeliac disease  Posterior uveitis  Autoimmune hepatitis  Atopic dermatitis  Autoimmune hemolytic syndrome  Crohn’s disease  Ulcerative colitis  IgA deficiency  Alopecia areata | 12 (31.6)  18 (47.4)  1 (2.6)  3 (7.9) 3 (7.9)  2 (5.3)  0 (0)  0 (0)  1 (2.6)  1 (2.6)  1 (2.6)  1 (2.6)  1 (2.6)  1 (2.6) | 5 (62.5)  1 (12.5)  0 (0)  0 (0)  0 (0)  0 (0)  1 (12.5)  1 (12.5)  0 (0)  0 (0)  0 (0)  0 (0)  0 (0)  0 (0) | 0.1036  0.0715  0.6484  0.416  0.4161  0.5101  0.0293  0.0293  0.6484  0.6484  0.6484  0.6484  0.6484  0.6484 |
| Immunodeficiency, n (%)  IgA deficiency  Hypogammaglobulinemia  LRBA deficiency | 1 (2.6)  0 (0)  1 (2.6) | 0 (0)  1 (12.5)  0 (0) | 0.6484  0.0293  0.6484 |
| Family history for AIG, n (%)  No  Yes  Unknown | 33 (86.4)  3 (7.9)  2 (5.3) | 7 (87.5)  1 (12.5)  0 (0) | 0.9346  0.6781  0.5101 |
| Family history for gastric neoplasia, n (%)  No  Yes  Unknown | 35 (92.1)  1 (2.6)  2 (5.3) | 7 (87.5)  1 (12.5)  0 (0) | 0.6781  0.2158  0.5101 |
| Family history for autoimmunity, n (%)  No  Yes  Unknown | 21 (55.3)  14 (36.8)  3 (7.9) | 6 (75)  2 (25)  0 (0) | 0.3090  0.5286  0.4161 |
| Concomitant autoimmune polyglandular syndrome, n (%)  No  Yes  Suspected | 33 (86.4)  4 (10.5)  1 (2.6) | 0 (0)  0 (0)  0 (0) | <0.0001  0.3428  0.6484 |
| Prior PPI therapy for >3 months, n (%) | 8 (21) | 1 (12.5) | 0.5856 |
| *H. pylori* status, n (%)  Negative  Positive  Eradicated  Unknown | 36 (94.7)  1 (2.6)  0 (0)  1 (2.6) | 7 (87.5)  0 (0)  1 (12.5)  0 (0) | 0.4594  0.6484  0.0293  0.6484 |
| Hyper-homocysteinemia, n (%) | 1 (2.6) | 0 (0) | 0.6484 |
| Factors leading to diagnosis, n (%)  GI impairment  Hematological impairment  Autoimmune screening  Family history | 7 (18.4)  23 (60.5)  16 (42.1)  1 (2.6) | 4 (50)  3 (37.5)  1 (12.5)  1 (12.5) | 0.0595  0.2381  0.1189  0.2158 |
| Previous misdiagnosis, n (%) | 12 (31.6) | 5 (62.5) | 0.1036 |
| Misdiagnosis, n (%)  Dyspepsia-other gastritis  Gastroesophageal reflux disease  Other form of anemia  Leukemia | 3 (7.9)  3 (7.9)  6 (15.8)  0 (0) | 3 (37.5)  0 (0)  0 (0)  1 (12.5) | 0.0255  0.4161  0.2331  0.0293 |
| Complication at onset, n (%) | 18 (47.4) | 4 (50) | 0.8947 |
| Type of complication at onset, n (%)  Pernicious anemia  Iron deficiency anemia  Gastric NETs | 1 (2.6)  19 (50)  1 (2.6) | 1 (12.5)  3 (37.5)  0 (0) | 0.2158  0.5246  0.6484 |
| Gastric NET, n (%)  No  Yes  Unknown | 36 (94.7)  1 (2.6)  1 (2.6) | 7 (87.5)  1 (12.5)  0 (0) | 0.4594  0.2158  0.6484 |
| Months from diagnosis to NET, median of months (IQR) | 84 (72-98) | 12 (9-18) | 0.002 |
| Total time of observation in moths, mean (SD) | 38.2 (11.24) | 40.8 (34.4) | 0.6988 |
| Total diagnostic delay in months, mean (SD) | 12.6 (34.1) | 10.5 (8.95) | 0.8646 |

Abbreviations: AIG, autoimmune gastritis; IQR, interquartile range; NET, neuroendocrine tumor; PPI, proton pump inhibitor; SD, standard deviation.
