# Supplementary material for: Pediatric autoimmune gastritis: An international, multicentric study
Source: J Pediatr Gastroenterol Nutr. 2025 Aug 12;81(5):1142–50. doi: 10.1002/jpn3.70187 (PMC12580456; doi:10.1002/jpn3.70187)
Supplement: Supplementary file 7 — Table S7. 08May25.docx. [file JPN3-81-1142-s007.docx]

**Supplementary Table 7**. Baseline and last available laboratory values based on anti-parietal cell antibody (PCA) status.

|  | **PCA positive** | **PCA negative** | **p value** |
| --- | --- | --- | --- |
| Vitamin B12, mean (SD) | 351.7 (210.89) | 389.9 (230.18) | 0.6487 |
| Folic acid, mean (SD) | 7.40 (5.44) | 7.7 (5.58) | 0.8884 |
| Ferritin, mean (SD) | 13.9 (13.97) | 13 (13.42) | 0.8684 |
| Gastrin 17, mean (SD) | 593.5 (977.64) | 342.6 (330.75) | 0.4804 |
| Chromogranin A, mean (SD) | 95.2 (170.23) | 126.4 (168.61) | 0.6393 |
| Last available gastrin 17, mean (SD) | 670.8 (800.81) | 339.4 (333.02) | 0.2598 |
| Last available chromogranin A, mean (SD) | 85.6 (144.81) | 88.7 (27.31) | 0.9526 |

Abbreviation: SD, standard deviation.
